# Review of Biomedical Applications of Contactless Imaging of Neonates Using Infrared Thermography and Beyond

**DOI:** 10.3390/mps1040039

**Published:** 2018-10-29

**Authors:** Abbas K. AlZubaidi, Yahya Ethawi, Georg M. Schmölzer, Sherif Sherif, Michael Narvey, Molly Seshia

**Affiliations:** 1 Biomedical Engineering Division, University of Saskatchewan, Campus Dr 9, Saskatoon, SK S7N 5A5, Canada; 2 Section of Neonatology, Winnipeg Regional Health Authority, Winnipeg, MB R3B 1E2, Canada; yethawi@hsc.mb.ca (Y.E.); mnarvey@hsc.mb.ca (M.N.); 3 Section of Neonatology, Department of Pediatrics, University of Alberta, Edmonton, AB T6G 1C9, Canada; georg.schmoelzer@me.com; 4 Department of Electrical and Computer Engineering, University of Manitoba, Winnipeg, MB R3T 5V6, Canada; Sherif.Sherif@umanitoba.ca; 5 Section of Neonatology, Department of Pediatrics, University of Manitoba, Winnipeg, MB R3A 1S1, Canada; mseshia@hsc.mb.ca

**Keywords:** preterm infants, NICU, neonatal imaging, physio-features, infrared thermography, optical coherence tomography, tissue optics, near-infrared, short-wave infrared, visible light

## Abstract

The sick preterm infant monitoring is an intriguing job that medical staff in Neonatal Intensive Care Units (NICU) must deal with on a daily basis. As a standards monitoring procedure, preterm infants are monitored via sensors and electrodes that are firmly attached to their fragile and delicate skin and connected to processing monitors. However, an alternative exists in contactless imaging to record such physiological signals (we call it as Physio-Markers), detecting superficial changes and internal structures activities which can be used independently of, or aligned with, conventional monitors. Countless advantages can be gained from unobtrusive monitoring not limited to: (1) quick data generation; (2) decreasing physical and direct contact with skin, which reduces skin breakdown and minimizes risk of infection; and (3) reduction of electrodes and probes connected to clinical monitors and attached to the skin, which allows greater body surface-area for better care. This review is an attempt to build a solid ground for and to provide a clear perspective of the potential clinical applications of technologies inside NICUs that use contactless imaging modalities such as Visible Light Imaging (VLI), Near Infrared Spectroscopy (NIRS), and Infrared Thermography (IRT).

## 1. Introduction

**Figure 1:**
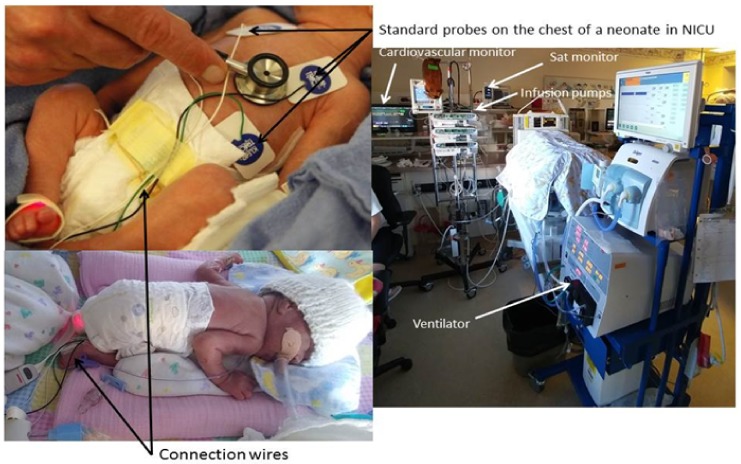
Standard monitors for a neonate inside a Neonatal Intensive Care Unit (NICU).

Contactless imaging can be used to obtain a variety of vital signs to assess the health of a subject. Examples of these vital signs include heart rate (HR), body temperature, respiratory rate (RR), blood pressure (BP), peripheral vascular resistance (PVR), and cardiac output (CO). For the purpose of this paper, these vital signs will be referred to as “Physio-Markers” (PM), and their individual characteristic patterns will be referred to as “Physio-Features” (PF). These markers would be the future vital signal indicators used inside Neonatal Intensive Care Units (NICUs) which currently suffer from crowded and entangled electrodes, sensors and devices as shown in Figure 1.

### 1.1. Significance to Emphasize the Importance of Contactless Imaging

**Figure 2:**
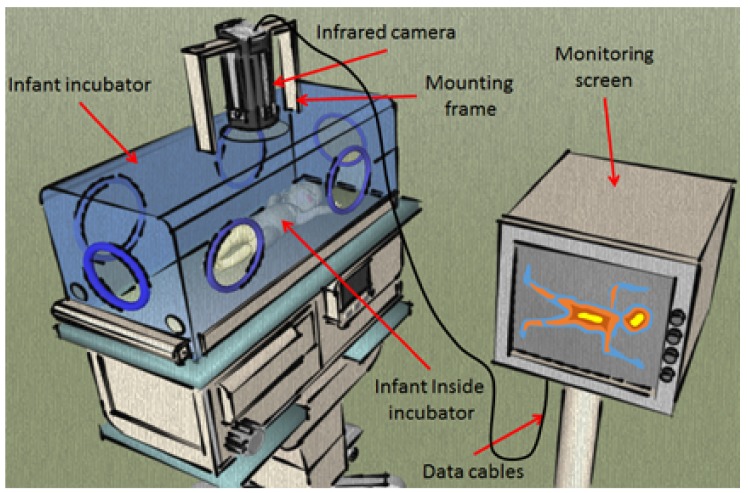
First contactless imaging setup used by Clark in 1980. This setup uses a traditional infrared camera with five frames per second (fps) and 5 ${}^{\circ }$C resolution.

Over the last three decades, contactless imaging in neonatal medicine has gained much attention, beginning with Clark’s and Sothers’ [1] first attempts to image preterm infants using infrared thermography. Clark’s experiment opened the door to a variety of applications for contactless imaging. The experiment itself was simple, consisting of a single infrared camera mounted vertically on an incubator’s Plexiglas hood to image the infant inside (see Figure 2). In the time since these first tests, several further investigations into contactless imaging’s potential have been conducted using visible light, infrared, and ultraviolet (UV) spectra [1,2].

**Figure 3:**
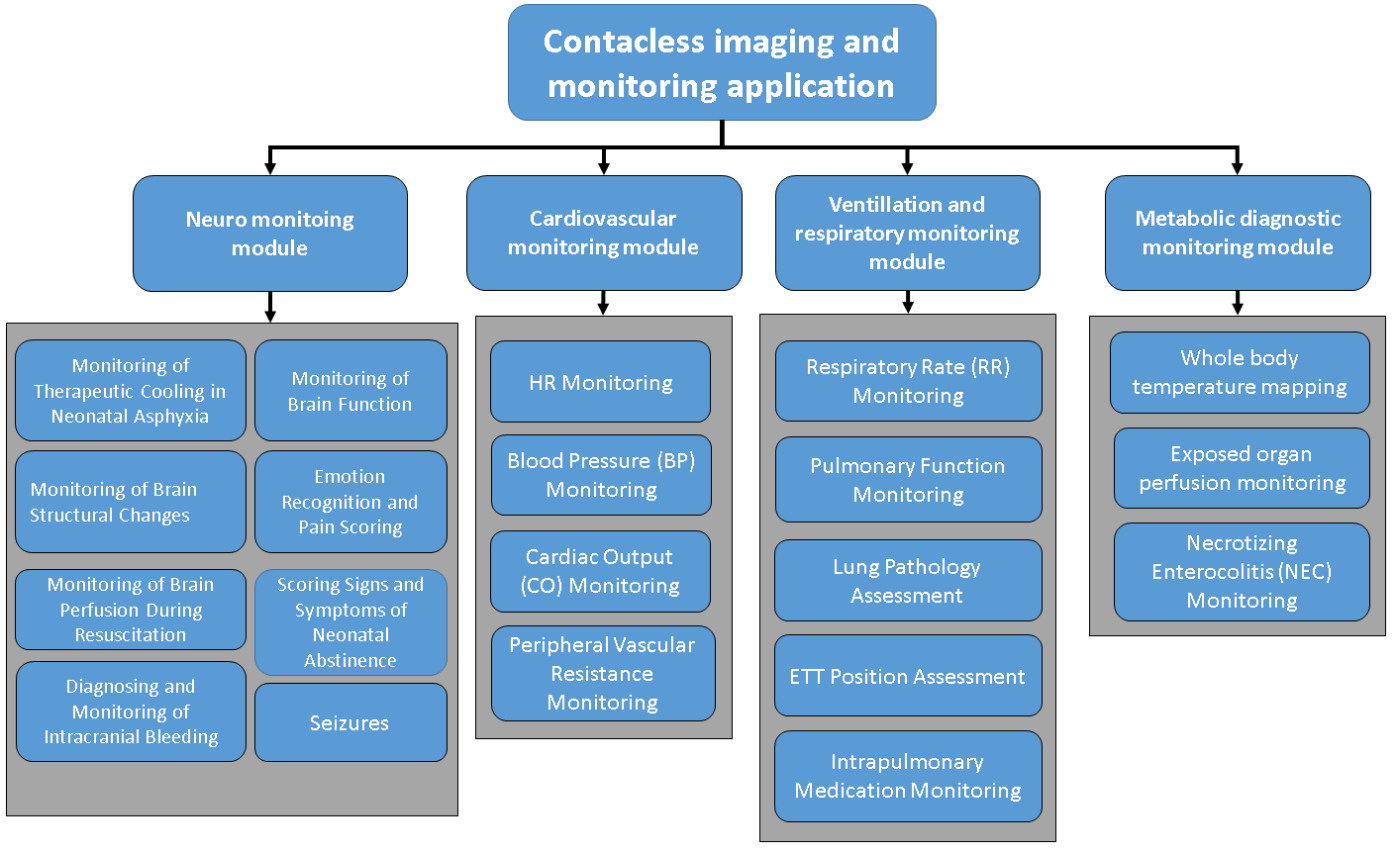
Potential clinical application of contactless imaging techniques, which can be widely used in neonatal intensive care unit (Source: [3]).

For general overview of what the potential application of these imaging modalities, Figure 3 demonstrates these clinical applications in neonatal medicine. Essentially, they are divided into four modules [3]: each module consists of several sub-applications according to the nature and needs of these imaging techniques.

The difference between contact and contactless imaging is relatively simple: contact imaging involves the direct application of an imaging sensor to the subject’s body (e.g., ultrasonography (US), electrical impedance tomography (EIT) [4,5], and bioimpedance tomography (BIS) [6,7]), while contactless imaging can detect, monitor, and visualize the subject’s anatomy or physiology without direct physical contact, particularly with the skin. As there is no physical contact with the skin, contactless imaging is considered safer and has fewer associated complications than contact imaging [8,9,10].

Developing new contactless technologies, and optimizing existing ones has the potential to create safer methods of diagnosing and monitoring preterm infants. In addition to being safer and having fewer potential complications, contactless monitoring may be a more cost-effective solution [11,12]. This article is part one of a two-part series, and it examines the technological aspects of contactless imaging approaches. The clinical applications of these technologies will be explored in part two, which is published in [3].

## 2. Contactless Neonatal Imaging Technologies

There are many contactless imaging technologies that may be potentially useful for solving different clinical problems. This section provides a non-exhaustive overview of example for most promising contactless technologies that may be useful in an NICU setting.

### 2.1. Electromagnetic Spectrum

**Figure 4:**
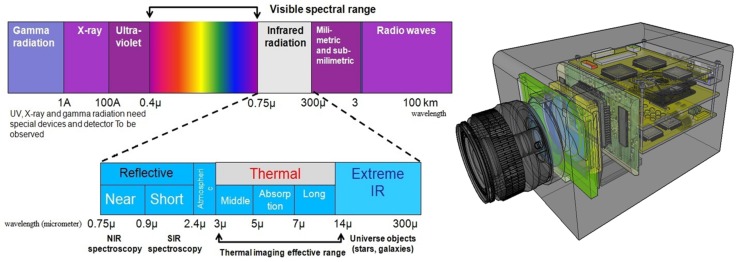
Electromagnetic (EM) spectrum showing infrared imaging band (reprinted with permission from one of the authors’ previous works [18]).

Before we dive into the contactless imaging technologies, we should touch the subject of electromagnetic (EM) spectrum and define it to formulate a solid ground for understanding the visible and infrared thermography imaging. EM spectrum can be defined as frequencies range of the EM radiation with respect to their photon energies and related wavelength [13]. This spectrum covers a wide range of frequencies ranging from sub-Hertz order to about 10${}^{25}$ of Hertz (see Figure 4). Each band assigned to convention based on its physical behavior starting from the end of these spectra (radio waves, microwaves) and followed by (teraherz waves), then by infrared and visible range (in which our review is focused on), then by ultraviolet (UV) and X-ray, then finally at the high frequency edge of this spectra (i.e., shorter wavelength) which is the (gamma rays) [14,15].

Infrared energy is part of the electromagnetic spectrum (see Figure 4) and has similar properties to visible light: it propagates through space at the speed of light, and it can be refracted, reflected, emitted, or absorbed [2,16]. However, infrared wavelengths (λ) are longer than those of visible light—which are between 0.7 and 1000 μm—and infrared energy is associated with thermal effects [13,17,18]. Essentially, the infrared spectrum can be divided into five distinct regions according to the wavelength which can be summarized with their respective wavelengths as follows: (i) Near-infrared (NIR) which has wavelength range between (0.7 μm–1.4 μm), (ii) Short-wave infrared (SWIR) which has wavelength range between (1.4 μm–3 μm), and (iii) Mid-wave infrared (MWIR) that has a range of wavelength between (3 μm–8 μm), but because of the atmospheric transmittance, which is approaching zero between (5 μm–8 μm), there is no detector (i.e., bolometer unit) operating in this infrared range; (iv) long-wave infrared (LWIR) which has a range of wavelength between (8 μm–15 μm) and (v) far infrared (FIR), which has a range of wavelength between (15 μm–1000 μm).

### 2.2. Infrared Thermographic Imaging

The mapping of human skin temperature, commonly known as thermal imaging technique and the data produced called thermal images and this technology is currently used in clinical practice and medical applications [15,19]. However, these thermal images need to be processed if they are to provide meaningful information for clinical practice.

The core temperature of the human body ranges between 36.5 ${}^{\circ }$C and 37.5 ${}^{\circ }$C [12,20], while the surface temperature is approximately 33 ${}^{\circ }$C [21]. Therefore, the range of human body temperatures that can potentially be recorded by a thermal (infrared) camera is between 33 ${}^{\circ }$C and 40 ${}^{\circ }$C, which is equivalent to a wavelength in the range of several micrometers (μm) [22].

Thermal imaging can detect different heat-energy-produced physiomarkers and physiofeatures that result from thermal-dynamic-induced events (cold or hot spots), for example, breast cancer [15,20] or local infections [23]. However, medical thermal imaging faces many challenges, such as standardized training to teach healthcare providers to interpret the images, and obtaining the necessary licensing from the appropriate regulating bodies—in addition to the periodic calibration of the thermal camera as well to decrease any error and certainties in the obtained thermographic data.

Table 1 presents the most recent and relevant work in the field of medical infrared thermography imaging covering the whole IR spectrum; these works resemble the stand-alone clinical and technical approach for integrating these technologies within the daily NICU workflow as part of smart neonatal incubator technologies.

NIR is an imaging modality that can map the oxygenation status of body tissues—especially high-oxygen-utilizing tissues such as those found in the brain and the bowel—to provide information about their metabolic and functional activities [9,16,18]. NIR imaging is not simple due to living tissue’s tendency to scatter light and also its anisotropic optical properties [29,37].

**Figure 5:**
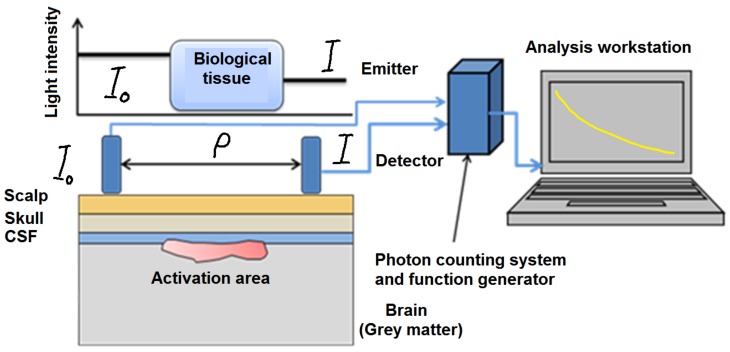
Basic mechanism illustration of the light absorption in NIR imaging of biological tissue. CSF: cerebrospinal fluid.

When the light propagates, it quickly diffuses throughout the tissue, as is shown in Figure 5. Therefore, it is not possible to image the internal absorbing structure by using a simple tomographic algorithm like those used in X-ray imaging [37]. Furthermore, the anisotropic structure will attenuate the light in a manner like absorption. Thus, it is difficult to differentiate the effect of absorption from the effect of scattering [14,38].

**Figure 6:**
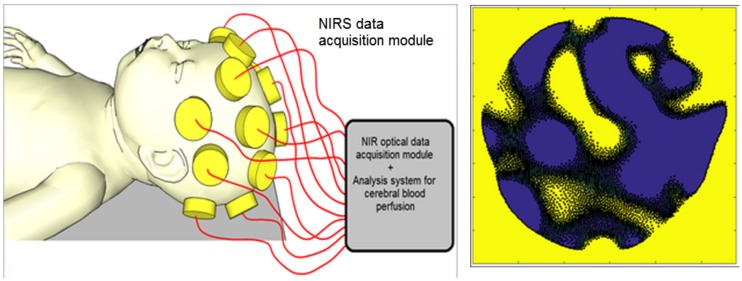
(**Left**) Diagram of neonatal NIR spectroscopy (NIRS) imaging. A series of photo-emitting diodes and photo-width = 0.9 are integrated to acquire NIRS data from multiple areas of the neonate’s head to reflect blood perfusion in the cerebral cortex. (**Right**) 2D reconstruction of NIRS images of a neonatal brain.

NIR imaging start with a pioneering modality known as optical topography, which uses a pair of single source-width = 0.9 (SSD) attached to the scalp’s surface to assess the function of a large surface area (SA) of the brain. However, this technique failed to provide adequate spatial resolution for imaging the internal structure of the brain [20,39,40,41]. Figure 6 shows another imaging modality, known as optical tomography, that generates two-dimensional (2D) or three-dimensional (3D) sectional images that are reconstructed to reflect the detail internal structure of the brain.

To date, topography and tomography have been applied to visualize the hemodynamics of the brain, with both being based on the measurement of flight times of photons traveling across the head. The distribution of photon flight times is unique and exclusive for each source-detector profile, and they provide sufficient information about the absorbance and scattering characteristics of light that is passing through the tissue.

The first 2D optical tomographic image of the brain was demonstrated by Benaron et al. [40] who developed an imaging system that measures photon flight times between various points on the head. Optical tomograms require multiple images, or slices of the brain, which are reconstructed using a straightforward back-projection method. This approach was used for detecting brain injuries in newborns, and could successfully demonstrate intracranial hemorrhages by detecting low oxygenated hemoglobin levels, which are a common characteristic of this condition.

A major drawback of this system is the simplicity of the image reconstruction algorithm, which ignores the inherent 3D nature of photon migration in tissues and the highly-complex nature of an infant’s head [42].

Another example of how NIR spectroscopy (NIRS) can be applied in a medical context is to examine changes in hemoglobin-oxygen relation to assess the functional activity of high-oxygen-consuming tissues, such as the cerebral cortex. In this application, an increase in afferent blood oxyhemoglobin (HbO) and a decrease in efferent blood deoxyhemoglobin (HbCO${}_{2}$) are associated with changes in the local cerebral blood flow (CBF), which reflects the local perfusion and the oxygen consumption [43,44,45].

### 2.3. Short-Wave Infrared Imaging

**Figure 7:**
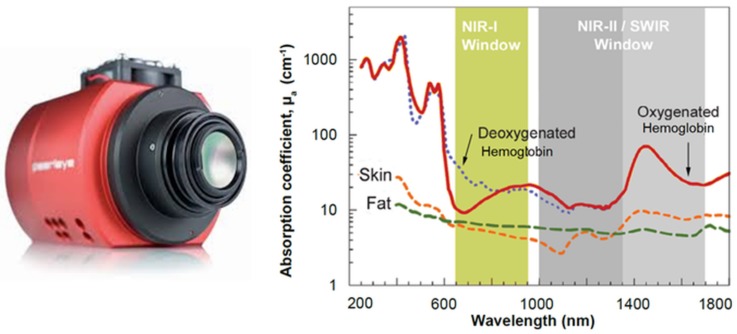
(**Left**) example of an SWIR camera used in research and medical applications (courtesy of Stemmer Imaging Inc, Zutphen, The Netherlands); (**Right**) absorption chart of light in tissue (courtesy of Dr. Dominik J. Naczynski, Stanford University, Stanford, CA, USA, adapted from [47]).

Principally, the SWIR wavelength (λ) band is located between 1 μm and 3 μm, which is also the band used for optical coherence tomography (OCT) imaging in tissues [46]. The SWIR wavelength band passes through the scattering medium and can penetrate deeper than visible or NIR wavelengths (see Figure 7).

Despite the importance of SWIR imaging in medicine, its usage is very limited in neonatal monitoring. The imaging of deep structures of biological tissues, which are optically opaque in nature, is the most challenging task for biological and clinical imaging.

Optical imaging with visible light provides high resolution and sensitivity; however, the scattering and absorption of the light by the tissue limit imaging depth to superficial structures.

Hence, imaging with SWIR shares many of the advantages with visible light imaging, but, unlike the last method, the scattering behavior of the tissue is reduced and significantly attenuated. Therefore, SWIR provides sufficient optical penetration depth to interrogate subsurface tissue features noninvasively [2,14,46].

Until recently, the potential applications of SWIR have been largely unexplored because suitable solutions have either been unavailable for clinical research or costly. However, new detector technology offers the opportunity to demonstrate how SWIR imaging can be used to improve clinical diagnostics. For example, SWIR scanning technology has been developed that can image the vascular system, and these images can provide valuable diagnostic information that can be used to complement the information obtained via Doppler ultrasound imaging, computerized tomography (CT) scans, or magnetic resonance imaging (MRI).

SWIR light’s ability to penetrate deeper tissue through the skin allows for better visualization of the human body, including the possibility of visualizing and detecting superficial vascular structures. Furthermore, SWIR’s potential for detecting small-vessel structures has not been well explored despite its potential usefulness for diagnosing complex vascular pathologies, such as hemangiomas and congenital vascular malformations [46].

It is important to know the effect of motion on the overall measurement, as the infant’s movement will affect the quality of acquired data, a motion-artifact compensation mechanism (e.g., detrending, polynomial interpolation) may be applied to the data avoiding any shifting or biasing in the SWIR data. Some severe effects may be observed if the infant’s motion are too gross and jerky in nature, which can lead to very low quality of SWIR data.

**Figure 8:**
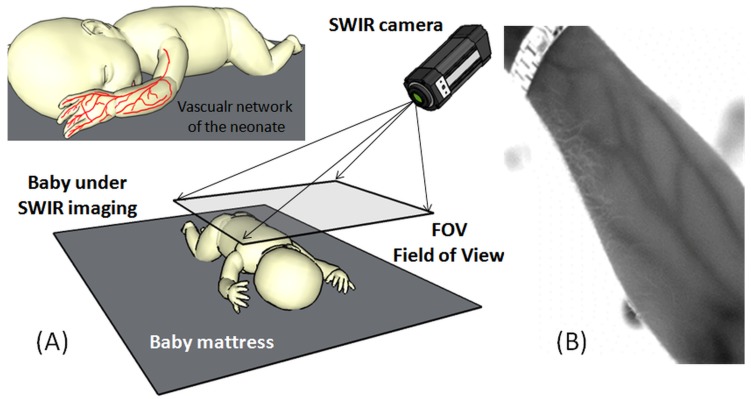
(**A**) imaging setup of the SWIR with field of view (FOV) set to cover the whole body of the neonate; (**B**) SWIR image of an adult hand showing the detailed vasculature of the forearm.

Figure 8 shows a typical SWIR imaging setup consisting of an SWIR camera positioned at a suitable distance (30 cm–60 cm) from the neonate to acquire the field of view (FOV) of interest. The camera can be used with an external light source to illuminate the region of interest and to detect the light reflection rate through the SWIR width = 0.9 [30,48].

Short waves spectrum has a limited ability to pass through the Plexiglas of infant incubator [1,19]. This limitation is due to the significantly degraded image (blurred, low resolution), low optical transmission rate (blurring of images associated with time) and temperature drift (inability of the camera to balance the difference between the temperature inside and outside incubator).

SWIR spectral range camera is perfect for neonatal imaging because the spectral band deviated from H${}_{2}$O transmission absorption region (6 μm–7.5 μm). Therefore, there is no need to put the SWIR camera inside the incubator or to use an optical window, such as a polyethylene (PE)-foil or another optical filter to image the neonate while they are cared inside incubator.

### 2.4. Middle-Wave Infrared and Long-Wave Infrared Imaging

There are several clinical implications within the range of Middle-Wave Infrared (MWIR) and Long-Wave Infrared (LWIR) region. The thermal spectral band lies between 3 μm–14 μm for MWIR and LWIR. Both have limited capabilities to pass through glass of the incubator due to degradation of the image (blurring). Therefore, the use of MWIR and LWIR are limited in neonatal clinical setting without the use of special optical windows (like: PE foils and infrared transparent optical materials) [9].

The selection of MWIR and LWIR clinical application are based on other factors like the body’s temperature distribution and dynamics, the circulatory system’s regulatory mechanisms (vasoconstriction, vasodilatation, auto-regulation, etc.), and the metabolic rates of different tissues [1].

The LWIR cameras are preferred for contactless imaging applications because it captures the absolute measurement (independent thermal point) or relative measurements (comparison of two points) of the thermal radiation of the newborn. The thermal energy of the newborn dominates other forms of the surrounding energy due to the characteristics of LWIR spectrum that not being present in other thermal spectral ranges (SWIR and MWIR).

Extreme care is required to ensure the radiometric measurement accuracy of the MWIR [13,49]. Finding a thermal reference during acquiring and converting MWIR thermal imaging is important as it provides thermal baseline points to limit detector-to-detector variation, which improves measurement accuracy. Rectangle, circle, triangle and sphere are examples of many thermal geometric references forms used for thermal reference purpose. These references may include sources with temperature-controlled extended area or uniformly-coated metal plates that have contact temperature sensors [18,50]. Although, for better calibration and temperature referencing, an industrial black body system can be used as well.

**Figure 9:**
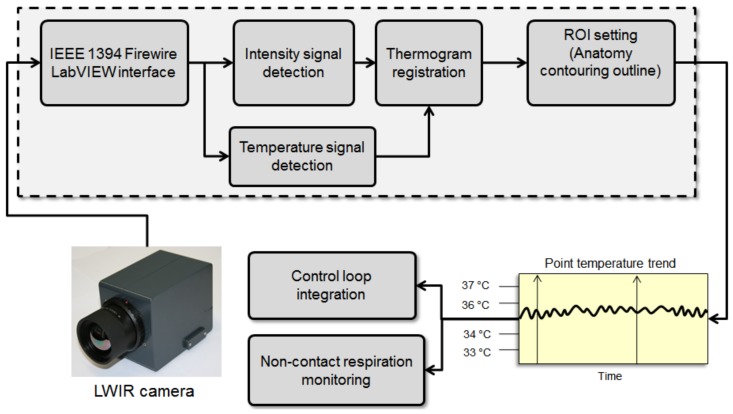
Block diagram of Long wave (LWIR)/middle wave infrared (MWIR) and short wave infrared (SWIR) thermography processing in a computer-aided diagnosis system.

Figure 9 shows the block diagram of a modern thermography acquisition system. This system starts with recording the temperature or thermal imaging points and then registers these points for the following steps. In these steps, several registered temperature points are used for the closed-loop control of the incubator, and temperature mapping and classification of thermal patterns of newborns.

Optical glasses with wavelengths (λ) ranging between 0.2 μm and 3.5 μm are transparent to light. This optical transmission then remains near zero level before the LWIR and very-long wavelength infrared (VLWIR) spectral regions [19].

There have been several attempts to use infrared thermal imaging to monitor the surface temperature of neonates.

**Figure 10:**
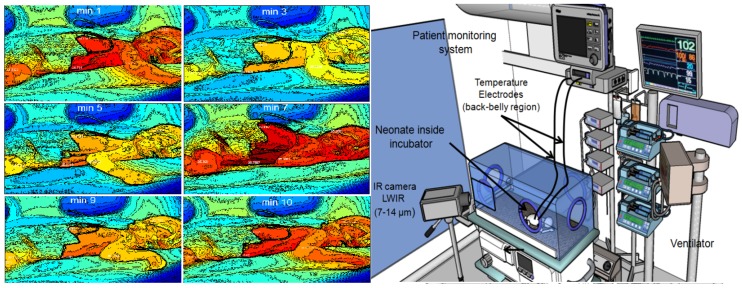
(**Left**) sequence of the neonatal thermograms showing the evolution of temperature distribution over time (photos reprinted from previous work of the author); (**Right**) typical experimental setup of neonatal infrared thermography (NIRT), including an Long wave infrared (LWIR) camera and associated vital sign monitoring inside an incubator (closed-type).

Figure 10 shows the Abbas et al. [19,24] trial who used an LWIR camera for skin temperature registration and monitoring in different clinical scenarios. Another practical trial, performed by Knobel et al. [25], used infrared thermography to examine the relationship between the evolution of necrotizing enterocolitis (NEC) and body-core temperature in premature infants [51,52].

In practical terms, infrared thermography is highly significant because it provides a solid foundation for several pathological and clinical methods of monitoring an infant’s body. For example, a surgical decision-making (SDM) module could be a significant aid in pediatric surgery. Monitoring in pediatric surgery requires dynamic revised updates based on assessments of the status and perfusion of the body regions being operated on. The SDM module reconstructs the images in real time to accurately reflect the extent of tissue necrosis for relevant body regions.

### 2.5. Photoplethysmography Imaging

Several investigators developed photoplethysmography imaging (PPGI) prototypes for testing and validation inside clinical setting [53]. The essential component of the PPGI system is a remote-based camera for visualizing superficial vascular networks and detecting blood volume changes in different areas of a measured object [42,54]. PPGI uses the same fundamental principle to improve on the poor spatial resolution inherent to conventional skin contact PPG. Therefore, the PPGI technique can be used as a tool for monitoring skin perfusion in newborn infants [55].

Measurements of the skin’s optical damping properties (0.4 μm to 0.7 μm of the visible spectrum and 0.4 μm to1 μm of near infrared) are dependent on blood and tissue composition within the measurement pathway between the light source and the detector [27,29,56]. This structure is modulated by changes in blood volume in the venous and/or arterial systems in various patient-activity states [57,58].

Conventional PPG sensors consist of one or more light sources, usually light-emitting diodes (LEDs), and either a phototransistor (PT) or photodiode light detector (PLD) [24]. Depending on how these components are positioned, measurements are conducted either in transmissive mode (e.g., in clips for earlobes or finger tips) or reflective mode (e.g., adhesive sensors on the skin surface).

PPGI measurements use a high-resolution camera with a multi-wavelength tissue illumination array instead of the singular contact sensor that is used today in pulse oximetry technology [59,60,61].

This measurement approach allows for contactless assessment of the tissue perfusion with the same spatial resolution as in the camera’s field of view (FOV) [24,28,62]. Thus, every pixel of the camera’s sensor array can be considered a discrete element of the PPG sensor, as the functional-imaging frame of the FOV assesses vital parameters by performing computation for predefined regions of interest (ROI) or each pixel separately [63,64].

The PPGI is suitable for measuring the same physiological parameters as PPG, including dynamic parameters [27,65,66]. Some examples of dynamic parameters include: capillary refill time, muscle pump efficacy, venous outflow, pulse characteristics (rate, variability, rhythm) [11,12,49], and arterial oxygen saturation. The main divergence between PPGI and PPG is in the composition of the measured light intensities and the number of elements used for detection [54,67].

The principal applications of PPGI are either simultaneous measurements of comparable locations in the body or inaccessible areas that require contact PPG sensors. One example of an instance where PPGI is useful for measuring inaccessible areas is in the assessment of skin perfusion reactions to allergy tests [48,65,68].

Additional potentially useful application of PPGI is to use a specially-mounted PPG camera, with its related lighting panel, inside a neonatal incubator to monitor allergy responses in the skin following a blood transfusion and other clinical interventional procedures [23,67,69].

Moreover, neonataologists can also use these technologies to assess the hypersensitivity allergic reaction and related immune system reactions, by using the blood perfusion signal as derived index for this type of reactions [27].

### 2.6. Neonatal Imaging with Ambient/Visible Light Variations

Information can be detected and processed using ambient light reflection, scattering, and diffusion in human skin. The interpretation of photoplethysmography data or visible intensity variations can provide an explicit representation of human tissues.

The data contains multiple projections of intensity changes, hue, saturation, and energy dissipation, which can provide additional information about the tissue being imaged. Several attempts have been made to develop an image/video processing algorithm for extracting and interpreting physical information related to the pixel value variation during the visible light video acquisition [36,41].

**Figure 11:**
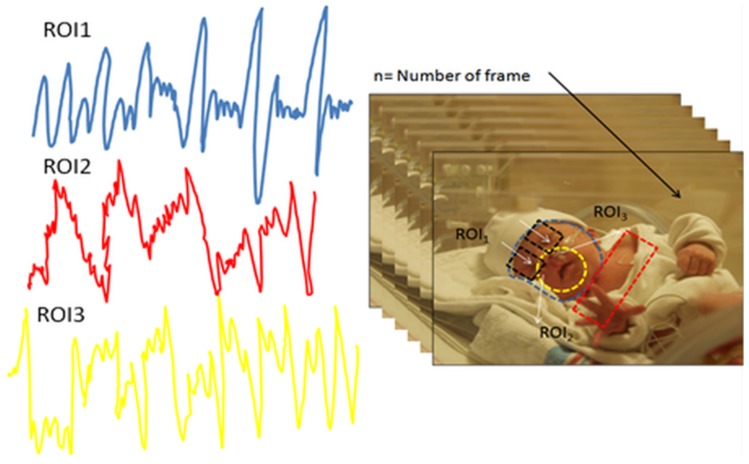
(**Left**) extracted gradient movement from defined regions of interest (ROIs) based on color intensity variation in successive video frames; (**Right**) video frame series of the neonate inside the incubator with three defined ROI profiles over forehead, nose, and shoulder.

Visible light imaging (VLI) is a robust and cost-effective method for analyzing the external cutaneous hemodynamic changes in the body. An example of these dynamics is observable in exposed skin vasculature where there is an insensible displacement recognized by rigid-motion detection within visible video recording as shown in Figure 11 (right) which can be used to detect pain, emotion, seizures and others. However, this method is hampered by the fluctuation of ambient light and the instability of subject during video recording, which superimposes a certain level of inaccuracy and noise onto the acquired signal as shown in Figure 11 (left).

### 2.7. Monitoring Behavior and Emotion of Neonates

Technologies that can monitor movements and other physiomarkers are promising tools for assessing a newborn’s emotional status. They can record the responses of newborn to pleasant or stressful stimuli, approximate pain scores, and observe any abnormal movements [35,70]. The only technique presently used to assess neonatal behavior and emotional response is electroencephalography (EEG), which is a broad and general method for detecting and classifying these complex patterns.

Alternatively, a group of light-emitting diodes can be attached to the upper and lower limbs of preterm infants and be recorded continuously by a remote workstation to assess skeletal-based behavioral patterns as we can notice in Table 2 that has many of the detection rates for such related behavioral patterns associated with somatic involvement of the preterm infants. Therefore, developing a new method for quantifying these reactions and responses could be quite advantageous in the assessment of infants for experiencing a variety of emotions including pain.

One of the proposed solutions for such a monitoring strategy is to use a visible camera to detect the pattern of local and global body movements. This monitoring strategy can be achieved by incorporating these detected movements into virtual mechanical-linkage models of the neonate.

**Figure 12:**
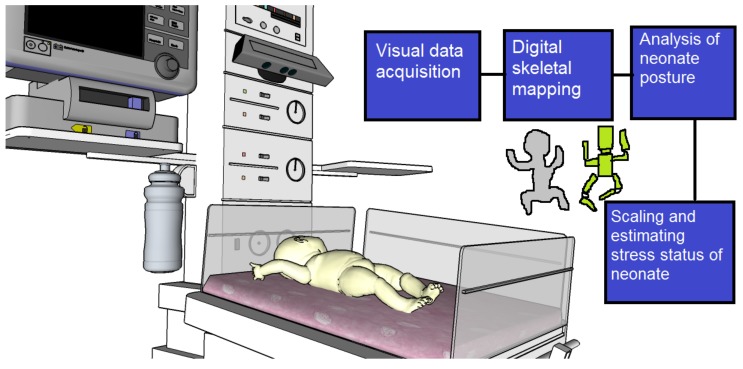
Microsoft Kinect experimental setup for mapping a neonate under intensive care, and estimation of behavioral patterns of the global and local body movements.

Converting these linkage models into meaningful biomechanical data can be a very useful method for assessing neonatal behavior and developing clinical applications. Figure 12 illustrates how a Microsoft Kinect® [24] device (Redmond, WA, USA) can be connected to the infant warmer to monitor and analyze the infant’s movements.

**Figure 13:**
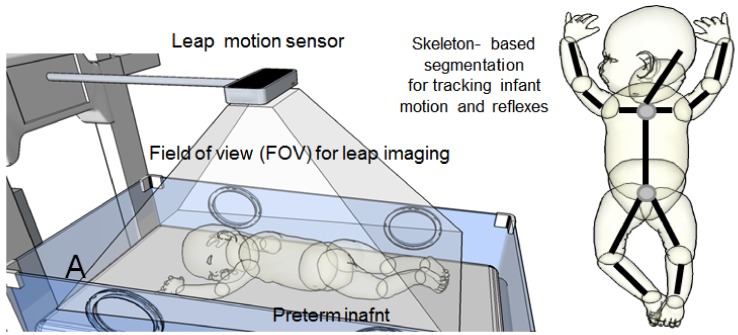
Experimental setup of a LeapMotion${}^{®}$ unit to monitor body movements of the neonate.

Another modality that can be used to track the neonate’s movements is leap motion for virtual reality (VR) (see Figure 13). Because the neonate can be as small as a human hand, leap motion can be a very powerful tool for detecting and tracking motion in newborn babies.

It is crucial to mention that the bundling of electrodes attached to the baby and positioning of him/her may affect this measurement and can make it inconsistent over the course of recording. Therefore, using several leap-motion devices positioned at different angles could solve this problem.

### 2.8. Computer Generated Graphics in Contactless Neonatal Imaging

As development of digital technology has accelerated, so too has the number of applications for computer graphics and computer-generated imagery (CGI). This boom has served to boost the potential application of these technologies in different fields, and the medical field is no exception. There has been great attention paid to these tools in the medical field, and they have become widely used in medical imaging (CT and MR), clinical educational simulators, and biomechanical analyses of human body.

This technology could be very useful for neonatal contactless imaging. Specifically, a high-speed camera could be used to image markers that are attached to the neonate’s body. These markers can then be segmented and processed in separate batches of image processing to identify several local/global regional movements—which will correspond to specific respiratory and pulmonary functions that the technology is intended to identify (Table 3).

**Figure 14:**
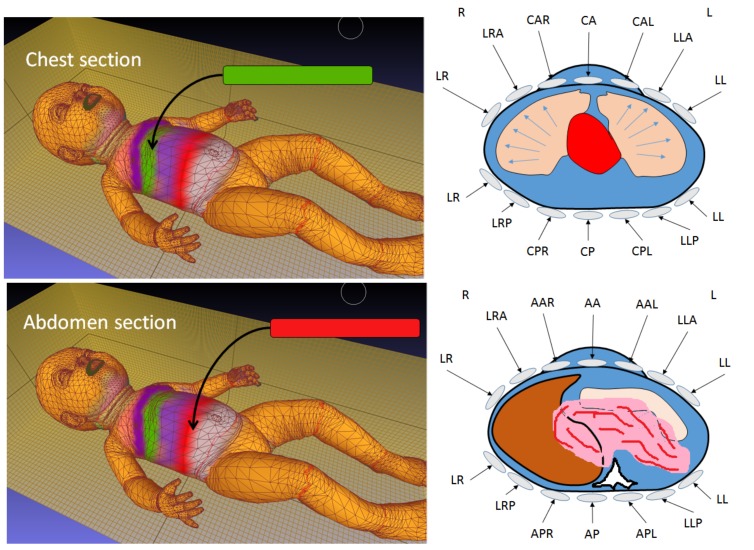
Experimental setup for CGI used in the detection and identification of respiratory and pulmonary functions by using a clinical inductive belt positioned at two levels (upper and lower) to generate several color-coded regions via CGI-vertex meshing colorization to indicate displacement and mechanical distortion due to several physiological- and pathological-related events.

Figure 14 illustrates the basic configuration of such CGI-generated images by using an inductive stretched respiratory belt that can be utilized to generate an image revealed the physiological activities associated with the change of spatial coordinate of these strips.

### 2.9. Embedding Contactless Imaging inside an Incubator

**Figure 15:**
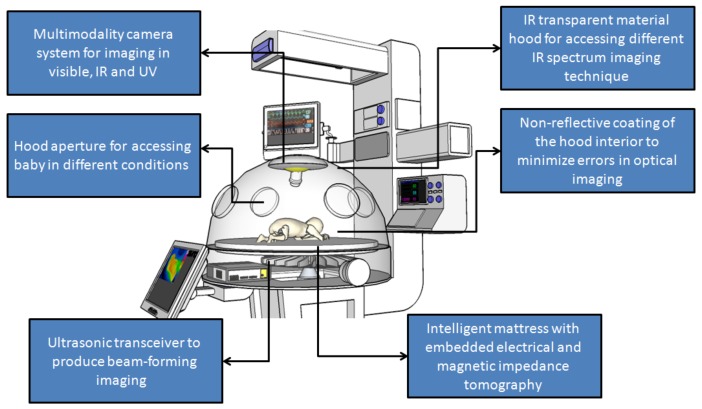
A schematic of neonatal incubator with multiple different embedded contactless imaging modalities for the early detection of various pathologies and neonatal diseases.

An example of an incubator that incorporates various contactless imaging modalities into its hardware can be seen in Figure 15. However, this incubator of the future faces one limitation: the level of specific absorption rate (SAR) for all these technologies in the same measurement space that may interfere with each other. This limitation may curtail the extent to which these imaging and diagnostic modules can be used with very small and fragile patients like newborn infants.

### 2.10. Infrared Thermal Tomography Imaging

This is a modified approach to the conventional thermal imaging; it is proposed to fully account for diffusion phenomena in a tomographic imaging proposition. Here, instead of the large area source used in conventional thermal imaging applications, a raster scanned point source is employed in order to provide the well-defined source-receiver positions required for tomographic imaging.

An algorithm for the forward propagation problem, based on the Pennes’ bioheat transfer (PBHT) method in combination with a Galerkin finite element method (FEM), these two mathematical models with the corresponding weak formulation for the thermal diffusion is considered. A thermal diffusion modified version of the algebraic reconstruction technique (ART) is used for this tomography approach.

**Figure 16:**
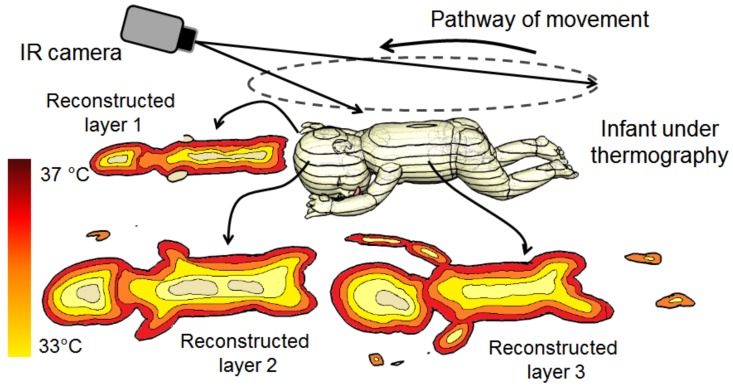
Imaging reconstruction of thermal tomography for neonates by using the dynamic thermal tomographic imaging (DYTTI) method; this gives the physician a clear representation of the temperature picture of the neonate’s body.

A Dynamic thermal tomographic imaging (DYTTI) method can be implemented and tested in the basis on inverse mathematical problem such as tomo- or topographic reconstruction method. A DYTTI is based on acquisition of the object under testing from different angles with time constant less than the 3 s, in order to catch the real-time surface temperature changes throughout the acquisition phase. Thus, providing multi-planar information about 75% of the infant’s body and therefore by using inverse thermal reconstruction, we can estimate the obscuring of the temperature profile of his/her body (see Figure 16).

Thermal tomography application in neonatology will give more quantitative bioheat modeling through multiple point temperature registration. This dynamic imaging method will open a door for more complicated clinical monitoring paradigm during neonate care (jaundice, hypothermia, sepsis, meningitis and other infectious diseases). Additionally, estimation of the internal profile temperature will give the physician a promising new tool for an early warning system of any complicated pathologies and disorders.

## 3. The Limitations and the Pending Challenges

Technically, there are several limitations that should be considered in applying a contactless imaging technique in the neonatal intensive care unit; most of these challenges and limitations are centered on the capability of reducing uncertainties and errors of the acquired thermal, visible imaging data and applying proper mathematical and signal processing methods before considering these technologies as potential tools in clinical practice.

Such limitation and challenges could be easy to solve and flexible compensation mechanisms can be realized and implemented to reduce them.

On the other hand, some of these challenges (e.g., temperature fluctuation, opacity of Plexiglas barriers, collateral thermal sources, uncalibrated cameras, and poor modulation transfer function (MTF) of bolometer detectors) are difficult to calibrate and need proper practical solutions to overcome such types of challenges.

Several errors are widely discussed by Abbas et al. [18], which covers the MWIR and LWIR imaging applications. Other applications treated in different views and perspectives can use these recommendations for further improvement of contactless imaging inside NICUs.

For the infrared thermal tomography imaging, several limitations can be accounted for related to this promising technology, such as the accuracy of a mathematical model that describes the detection points of the temperature propagation along the tissue axes, reference anatomical model of the infant cross-sections; the latter may need full body high resolution MRI or CT scans for a preterm infant used as a reference to compute the related superficial temperature points using inverse-point reconstruction problems.

## 4. Discussion

Contactless neonatal imaging can assert itself as a useful medical tool in the near future if the associated technological and clinical challenges can be addressed. Indeed, contactless imaging is not far from becoming a standard practice in NICUs. The parameters acquired from optical, NIR, SWIR, MWIR and LWIR, and other modalities will be invaluable if they are interpreted in a manner that facilitates more effective clinical decision-making in the NICU. Combining two imaging approaches (for example, one passive, like LWIR, and one active, like SWIR) can cover a wider range of physiomarkers and physiofeatures in clinical diagnostics and telehealth systems. Moreover, other computer imaging techniques can be used in conjunction with these contactless technologies. As computer imaging advances alongside contactless imaging, the coordinated use of these technologies (e.g., CGI, motion capturing, 3D augmented reality, etc.) can be helped to produce better contactless imaging and diagnosis of newborn infants and accelerate the future integration of these technologies in the upcoming NICU medical revolution.

## 5. Future Directions

In a prescriptive view, all of the above-mentioned technologies can be incorporated into futuristic design of the neonatal incubator, which could be the “state of the art” for different imaging, diagnosis and therapeutic modalities that could save millions of lives worldwide and can be the technology motivator for further development of human thermo-physiology management applications e.g., in deep space missions, patients with comas and clinical complications, where long-term monitoring is a vital aspect. Additionally, we can see the future of neonatal incubators well defined with these technologies as this will increase the productivity of medical staff by reducing human errors and monitoring interruption by using sophisticated imaging technologies fused with computer visions, artificial intelligence and machine learning algorithms. One of the possible directions of the contactless imaging technologies is developing an early-warning system for sepsis complication and infections by different pathogens and infectious sources.

## Figures and Tables

**Table 1:** Overview of research using imaging modalities.

Approach/Technologies/Methods	Related Work/Research Papers	Potential Clinical Applications
Infrared Thermography in Neonatal Thermoregulation	(Abbas & Leonhardt) [24] (Heimann) [9,24] (Knobel, Guenther, & Rice) [25], (Saxena & Willital) [26]	Temperature & metabolism monitoring, peripheral blood perfusion, respiration detection
Near Infrared (NIR) Imaging	(Blanik, Andropoulos, Arridge) [27,28,29]	Blood perfusion, Oxygen Saturation, cerebral blood flow, hemodynamic monitoring, brain injury, biomarkers imaging
Short Wave Infrared Imaging (SWIR)	Langston [30], Dong [31]	Peripheral blood perfusion, blood vessels imaging.
Middle and Long Wave Infrared Imaging (MWIR, LWIR)	(Abbas & Leonhardt) [24], (Allen) [32], (Gade & Moeslund) [33]	Stress imaging, emotional classification, monitoring of temperature related physiology, and thermal imaging of the nostrils
Photoplethysmography Imaging (PPG)	(Blanik) [27]	Hemodynamic monitoring, oxygen consumption monitoring.
Neonatal Imaging with Ambient/Visible Light Variation	(Nakamura) [34]	Monitoring of physiological signal such as heart rate, peripheral blood pressure and blood perfusion.
Embedding Contactless Imaging inside Incubator	(Abbas) [19]	Embedding of several physiological monitoring and imaging techniques inside the neonatal incubator such as (IR imaging, Magnetic impedance tomography (MIT)
Behavior and Emotional Monitoring of Neonate	(Gunnar) [35] (Vandenberg) [36]	Long-term monitoring and assessment of behavioral patterns of the neonates during daily care.

**Table 2:** Tabulation of the behavioral index used in the classification of neonate’s movement using infrared (IR)-kinect detector.

Behavior Index	Detection Head.%	Detection Upper Extem.%	Detection Lower Extem.%
BH1	78	90	91
BH2	62	92	73
BH3	79	96	95
BH4	71	86	93
BH5	86	93	82

**Table 3:** Physiological parameters that can be inferring from computer-generated imagery (CGI) neonatal imaging methods.

Parameter	Dynamic Range	Error Percentage %
Respiration rate (RR)	10–14	0.5–1.4
Heart rate (HR)	40–85	0.2–1
End tidal volume (ETV)	20–50 mmHg	3–5
Lung compliance (LC)	70–90 mL/cm H${}_{2}$O	3–6.2
